# Supplementary material for: Second Generation Small Molecule Inhibitors of Gankyrin for the Treatment of Pediatric Liver Cancer
Source: Cancers (Basel). 2022 Jun 22;14(13):3068. doi: 10.3390/cancers14133068 (PMC9265042; doi:10.3390/cancers14133068)
Supplement: Supplementary file 1 [file cancers-14-03068-s001.zip › cancers-1728297-supplementary.pdf]

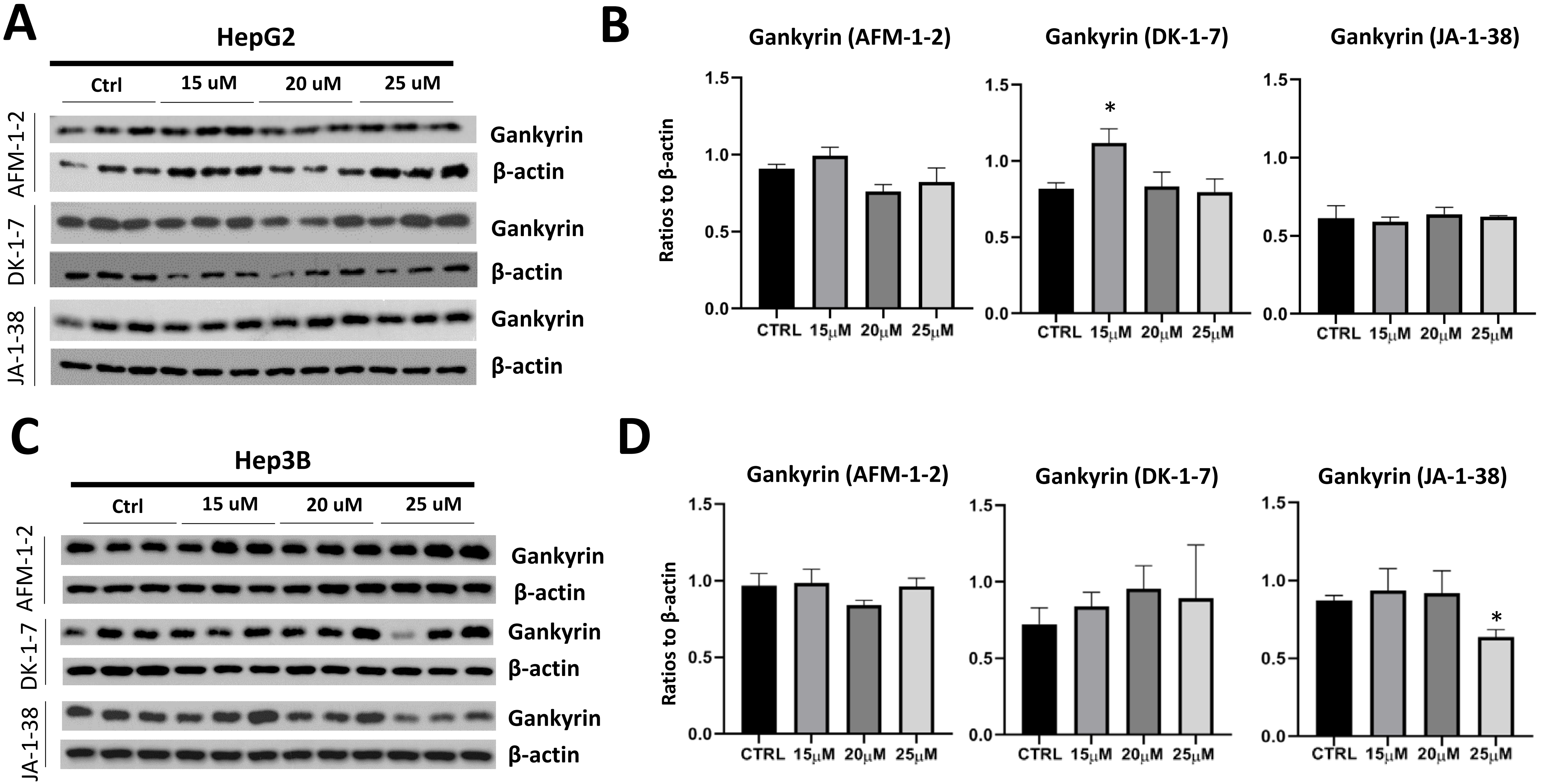

Figure S1. A. Western blot of Gankyrin expression in HepG2 cells treated with all three compounds. B. Quantitation of western blots of from Supplemental Figure S1A. C. Western blot of Gankyrin expression in Hep3B cells treated with all three compounds. D. Quantitation of western blots from Supplemental Figure S1C.

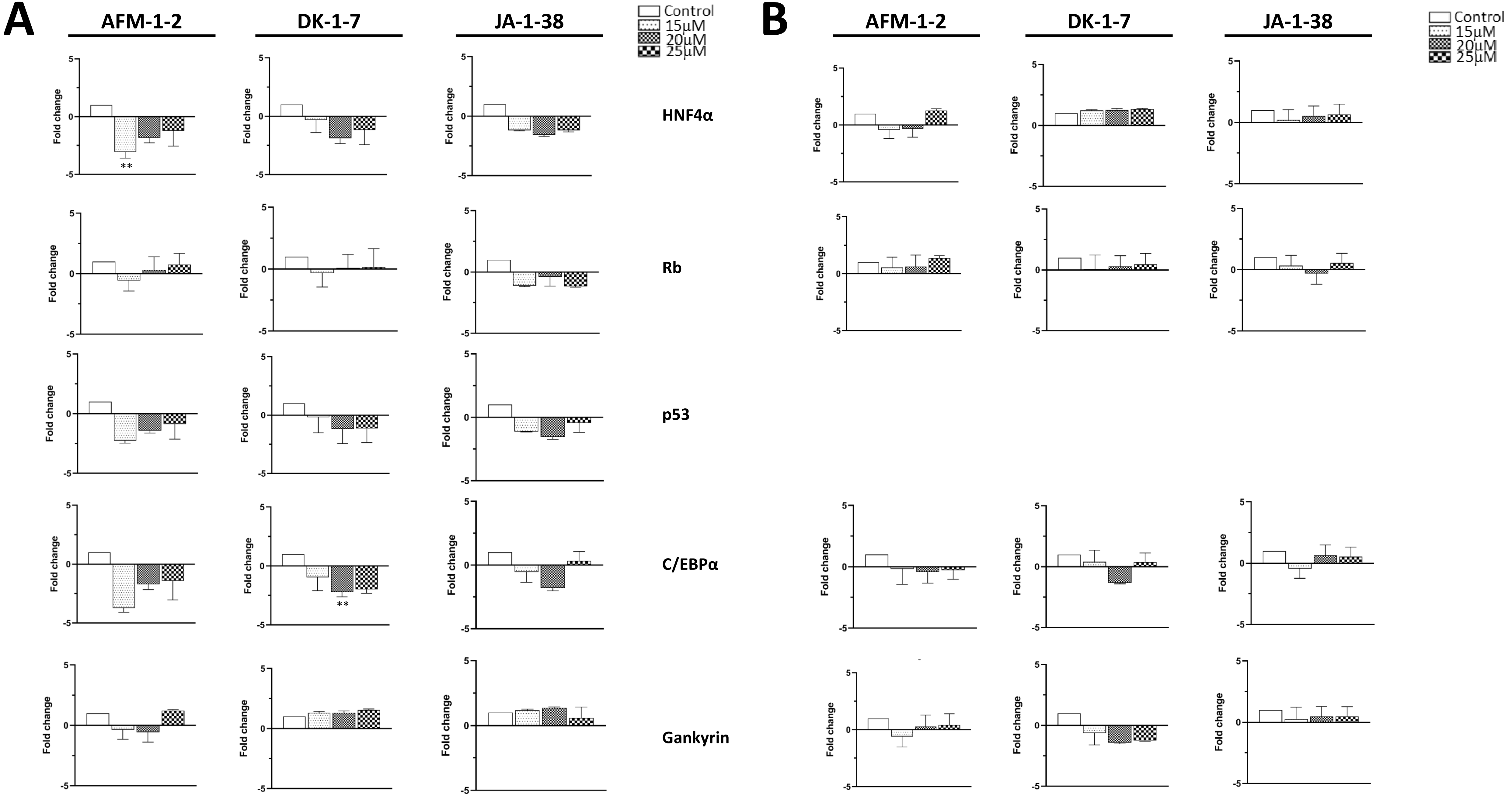

Figure S2. A. QRT-PCR results of HepG2 cells treated with AFM-1-2, DK-1-7, and JA-1-38. . In AFM-1 and -2, there was a statistically significant decrease in HNF4α at 15 μM ($p = 0.002$), and in DK-1–7, there was a statistically significant decrease in c/EBPα at 20 μM ($p = 0.003$). B. QRT-PCR results of Hep3B cells treated with AFM-1-2, DK-1-7, and JA-1-38.

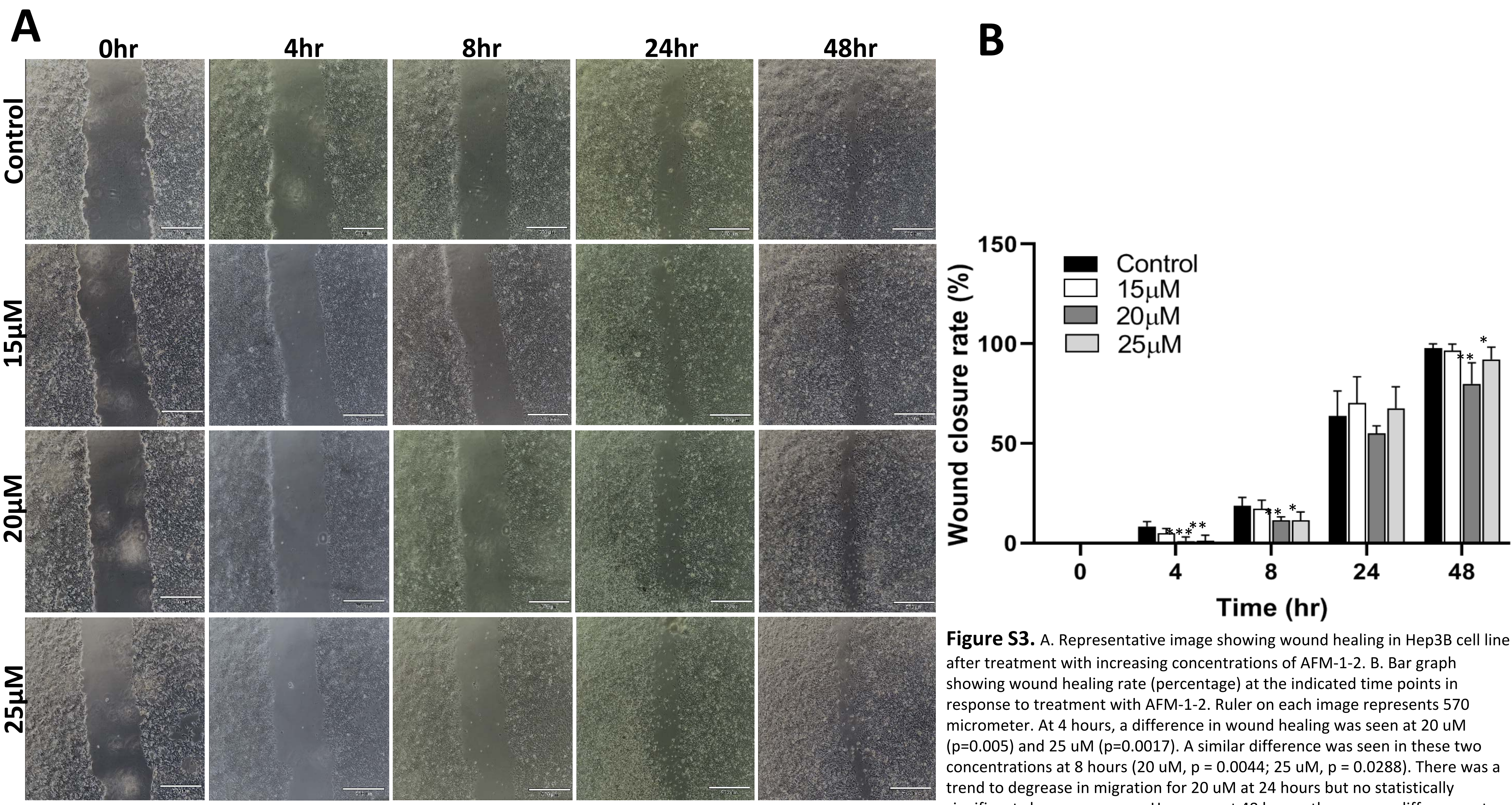

Figure S3. A. Representative image showing wound healing in Hep3B cell line after treatment with increasing concentrations of AFM-1-2. B. Bar graph showing wound healing rate (percentage) at the indicated time points in response to treatment with AFM-1-2. Ruler on each image represents 570 micrometer. At 4 hours, a difference in wound healing was seen at 20 uM (p=0.005) and 25 uM (p=0.0017). A similar difference was seen in these two concentrations at 8 hours (20 uM, p = 0.0044; 25 uM, p = 0.0288). There was a trend to degrease in migration for 20 uM at 24 hours but no statistically significant change was seen. However, at 48 hours, there was a difference at both 20 uM (p = 0.0035) and 25 uM (p = 0.0125) dosing.

Primer name	Sequence (5` to 3`)
HPRT-F	TGACACTGGCAAAACAATGCA
HPRT-R	GGTCCTTTTCACCAGCAAGCT
PSMD10-F	GGGTGTGTGTCTAACCTAATGG
PSMD10-R	GGCCAGAATACTCTCCTTCAACT
RB1-F	TTGGATCACAGCGATACAAACTT
RB1-R	AGCGCACGCCAATAAAGACAT
CUGBP1-F	CTGAAAAGGACTTGCGGGAAC
CUGBP1-R	GGCGGGTTTTGGCTCCTAT
HNF4a-F	CGAAGGTCAAGCTATGAGGACA
HNF4a-R	ATCTGCGATGCTGGCAATCT
C/EBPa-F	AGGAGGATGAAGCCAAGCAGCT
C/EBPa-R	AGTGCGCGATCTGGAACTGCAG
cdc2-F	GGATGTGCTTATGCAGGATTCC
cdc2-R	CATGTACTGACCAGGAGGGATAG
Epcam-F	TGATCCTGACTGCGATGAGAG
Epcam-R	CTTGTCTGTTCTTCTGACCCC
Thy1-F	CCTTCAGCACCTAAGTCCATAC
Thy1-R	GTGGACAATCCAGTCCAGAAA
CD13-F	GTTCTCCTTCTCCAACCTCATC
CD13-R	CTGTTTCCTCGTTGTCCTTCT
KRT19-F	AACGGCGAGCTAGAGGTGA
KRT19-R	GGATGGTCGTGTAGTAGTGGC
CD133-F	AGTCGGAAACTGGCAGATAGC
CD133-R	GGTAGTGTTGTACTGGGCCAAT
P53-F	TCAACAAGATGTTTTGCCAACTG
P53-R	ATGTGCTGTGACTGCTTGTAGATG
Oct4-F	GGGAGATTGATAACTGGTGTGTT
Oct4-R	GTGTATATCCCAGGGTGATCCTC
CD44-F	CTGCCGCTTTGCAGGTGTA
CD44-R	CATTGTGGGCAAGGTGCTATT

Figure S4

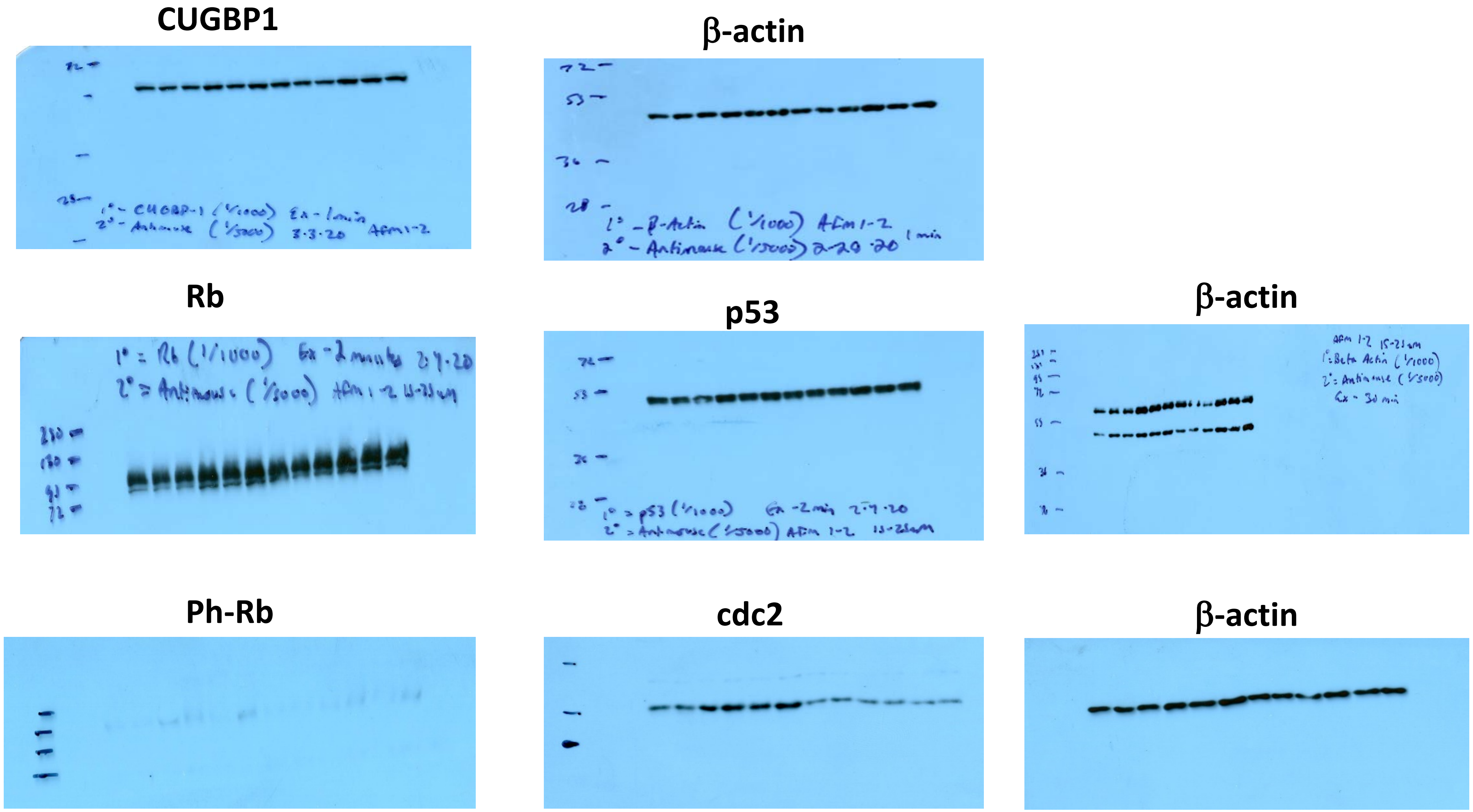

Original Western blot Images: Figure 3A

CUGBP1

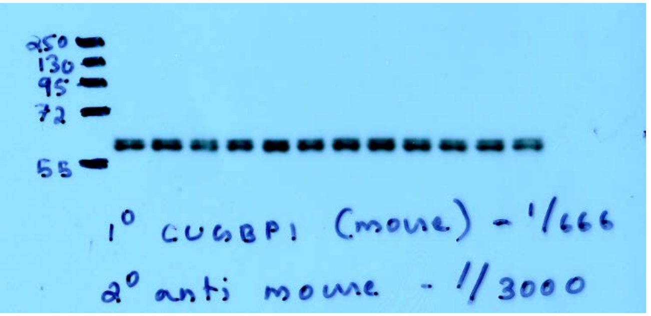

β-actin

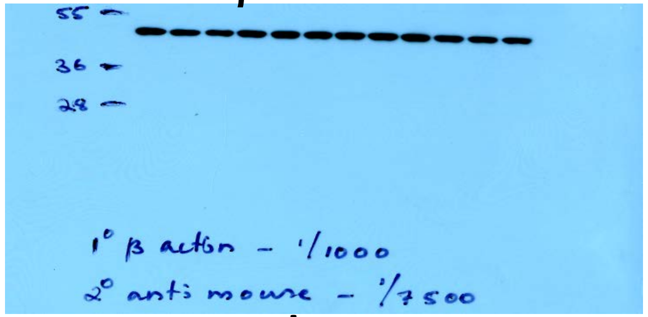

HNF4α

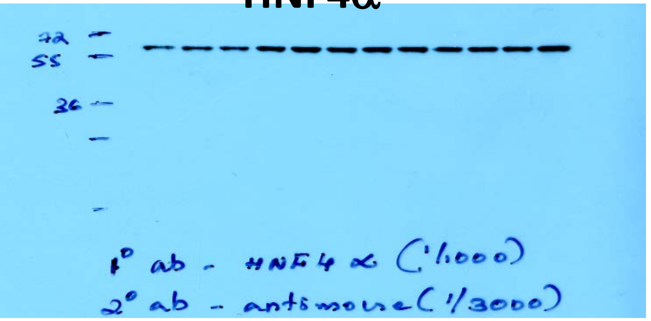

Rb

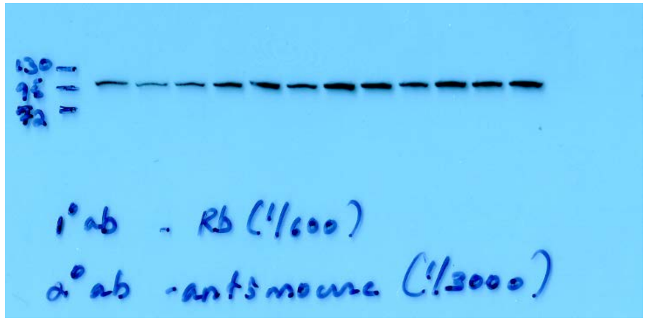

cdc2

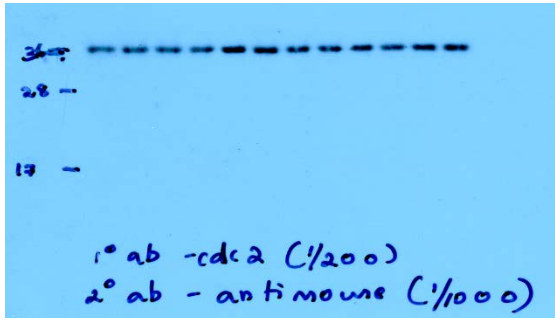

β-actin

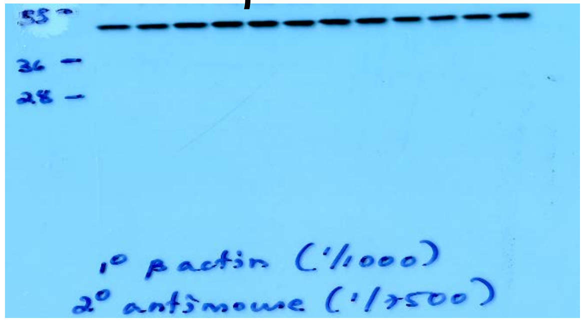

Ph-Rb

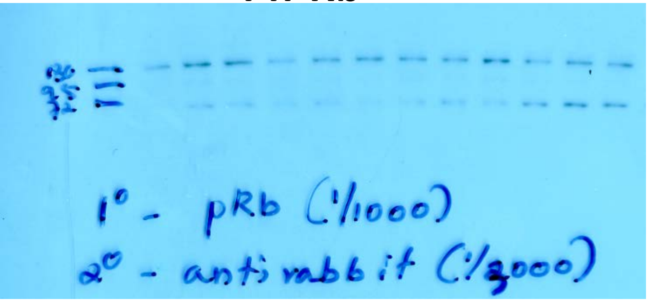

β-actin

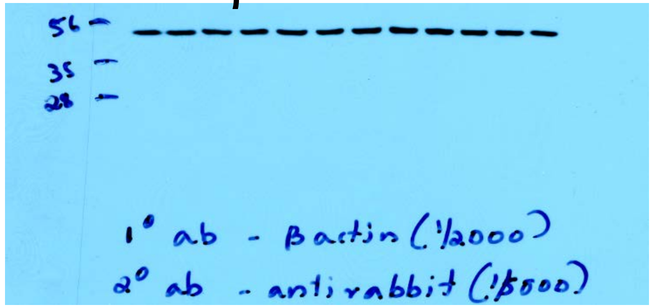

Original Western blot Images: Figure 3C

CUGBP1

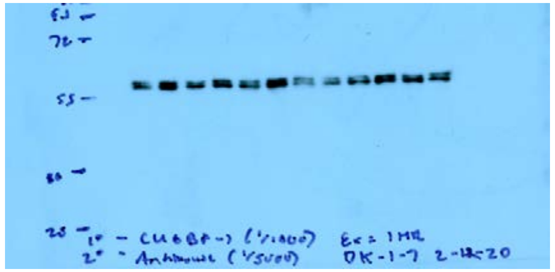

p53

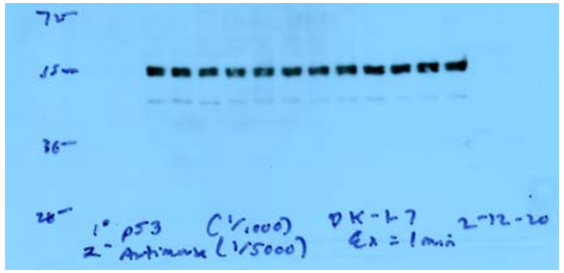

Rb

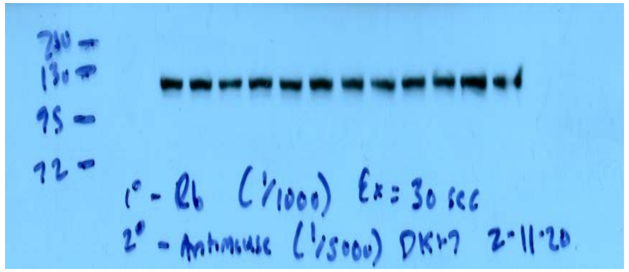

HNF4α

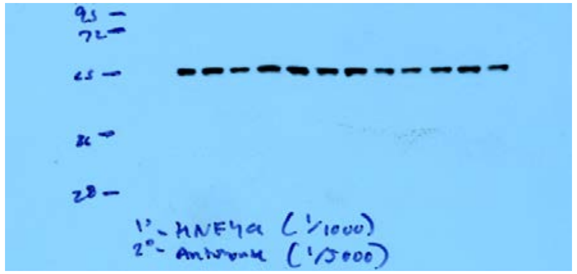

β-actin

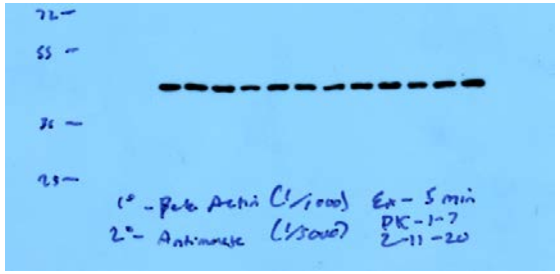

Ph-Rb

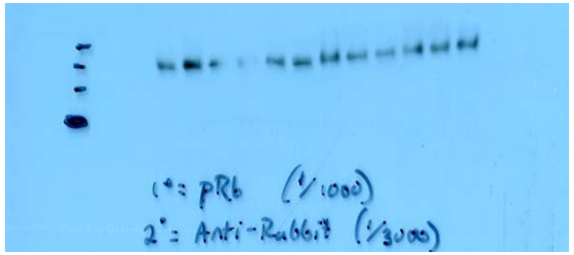

cdc2

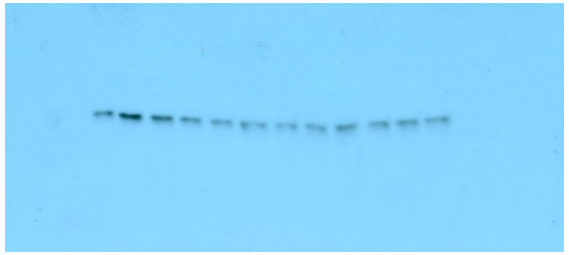

β-actin

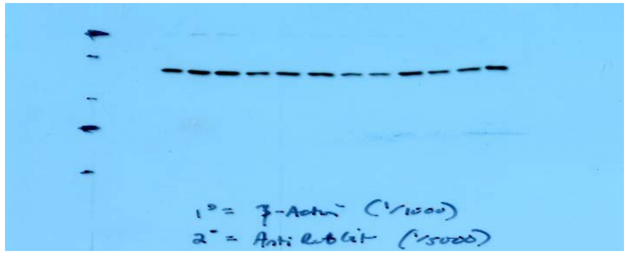

Original Western blot Images: Figure 4A

CUGBP1

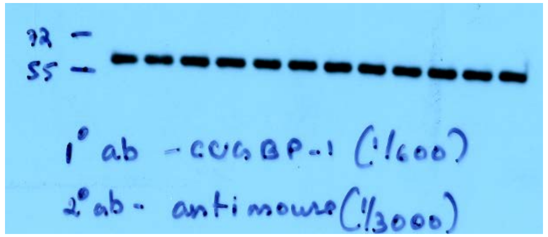

Rb

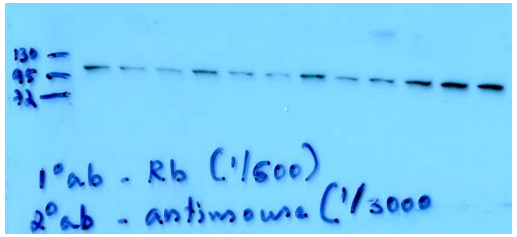

cdc2

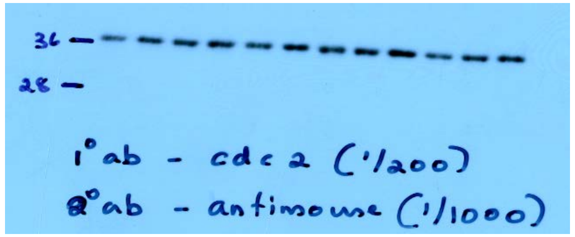

β-actin

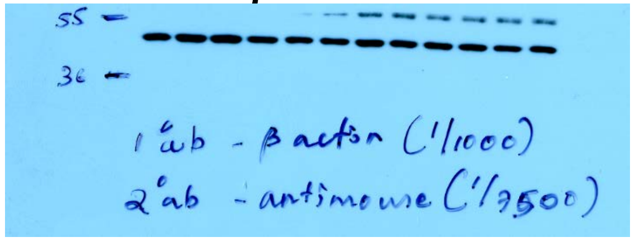

HNF4α

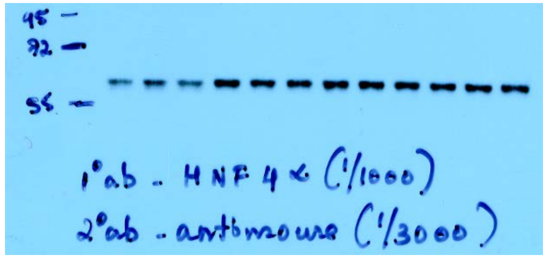

β-actin

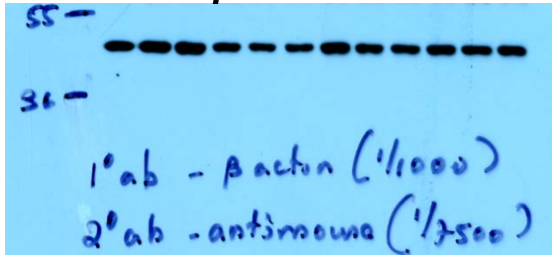

Ph-Rb

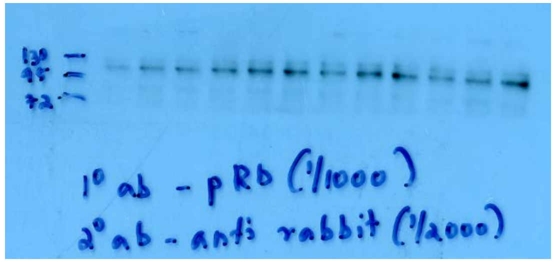

β-actin

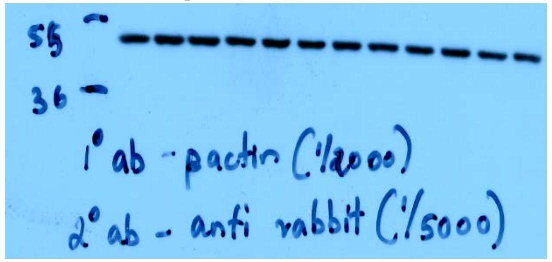

Original Western blot Images: Figure 4C

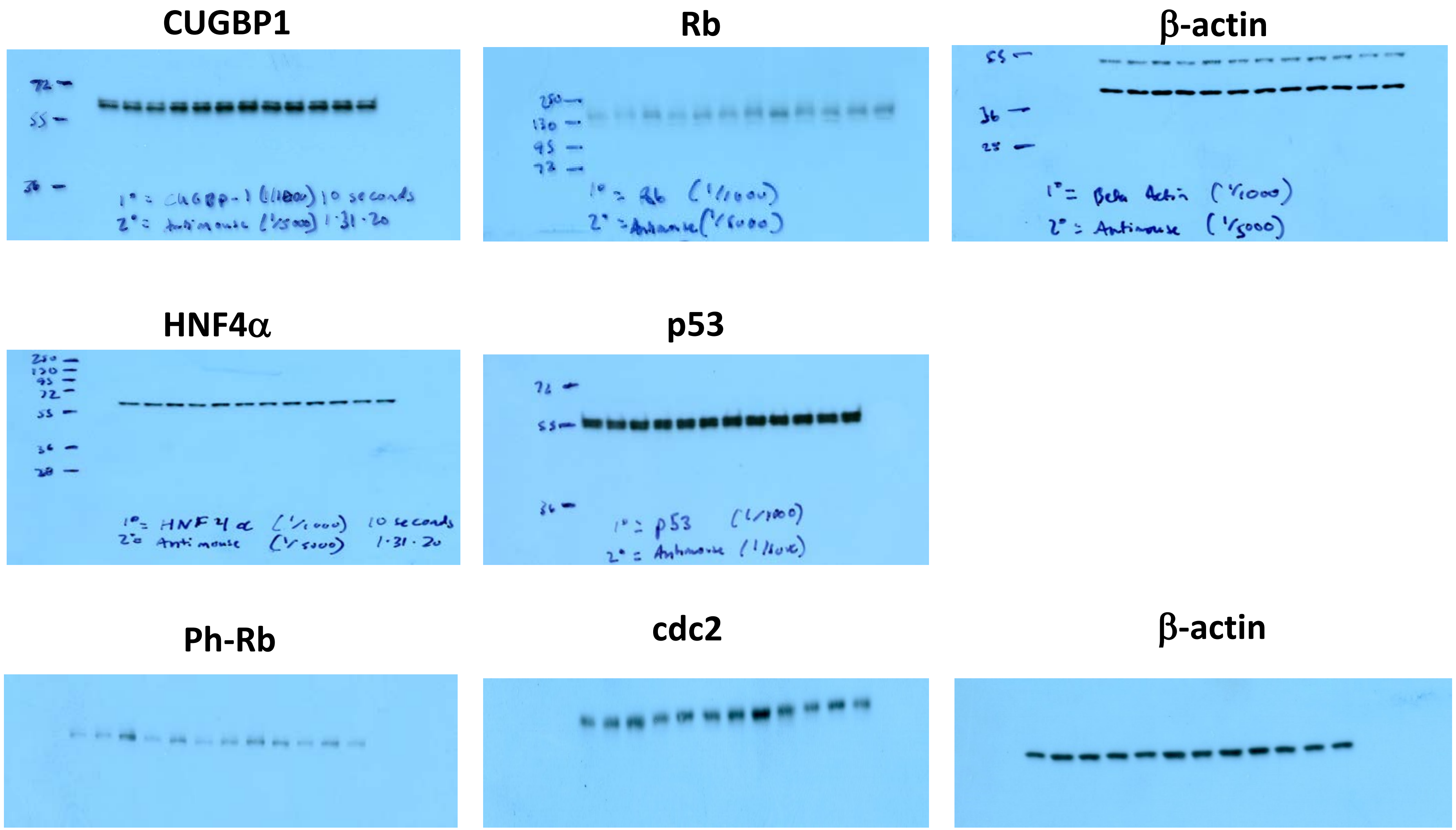

Original Western blot Images: Figure 5A

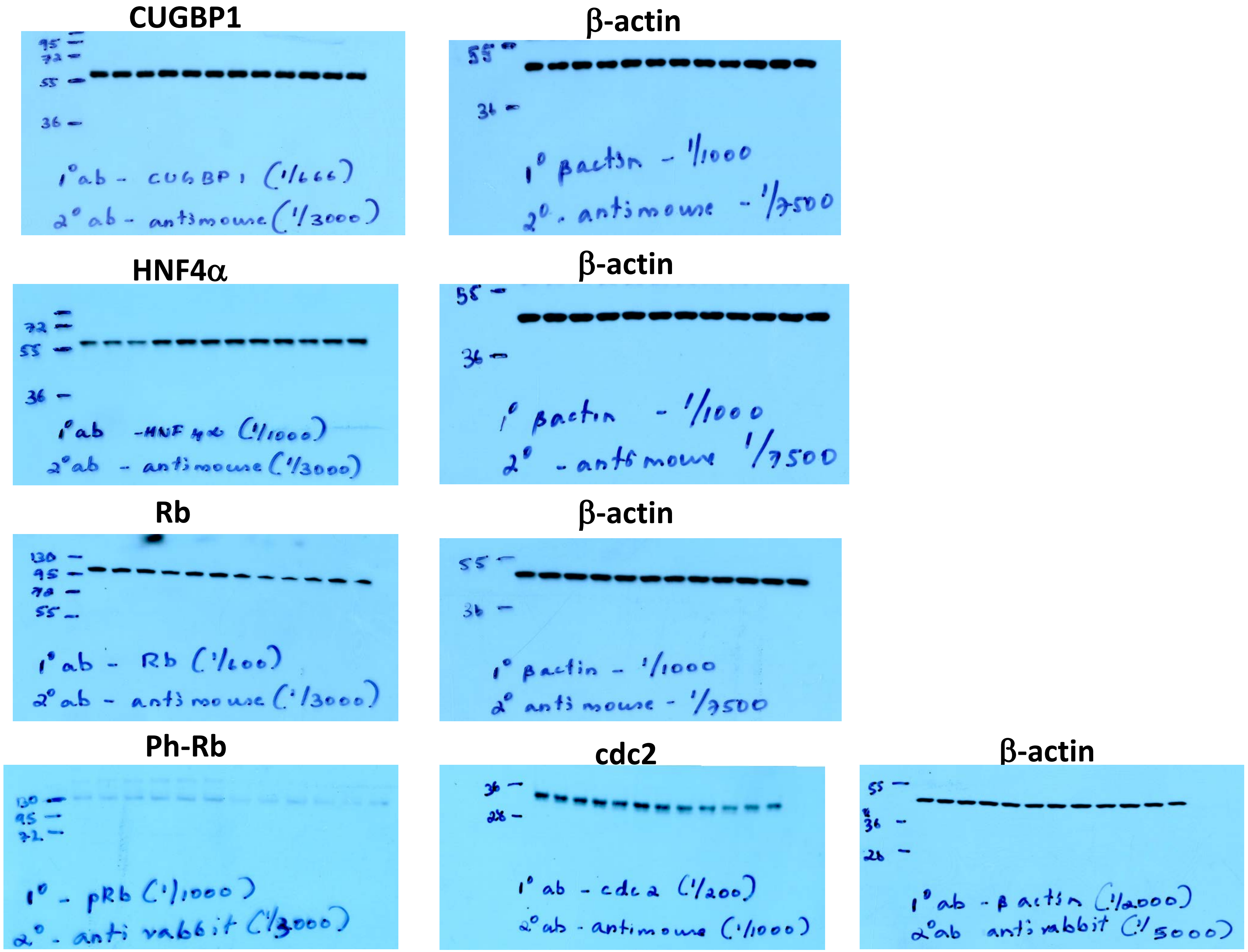

Original Western blot Images: Figure 5C

Proteasome α CYTO

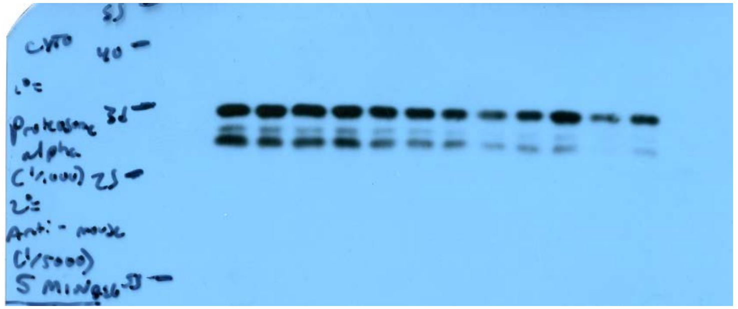

Proteasome α NUC

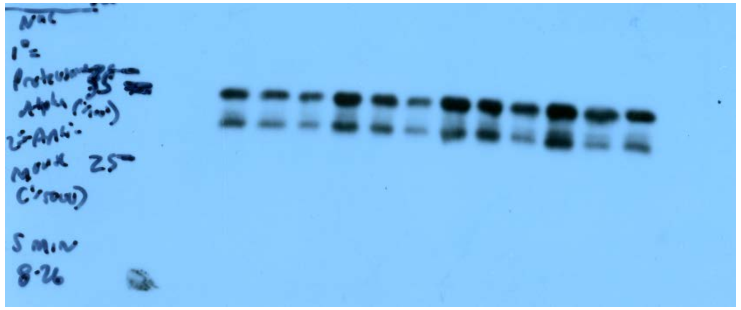

β-Actin

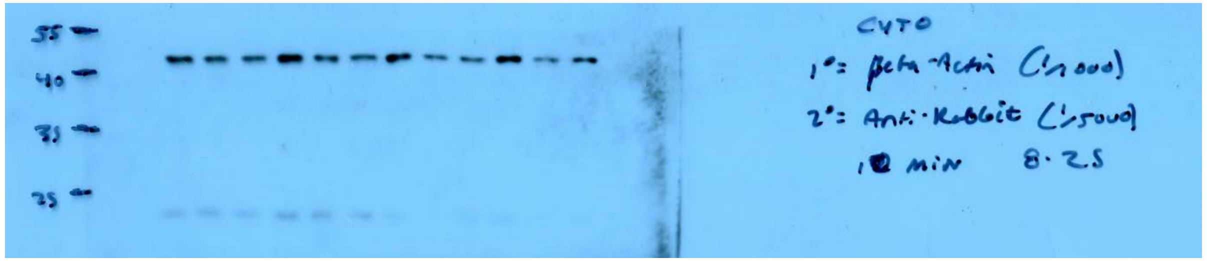

HDAC

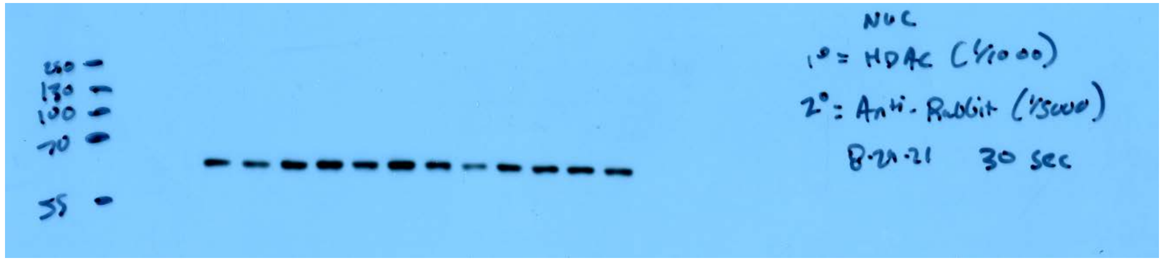

Original Western blot Images: Figure 9A

Proteasome α CYTO

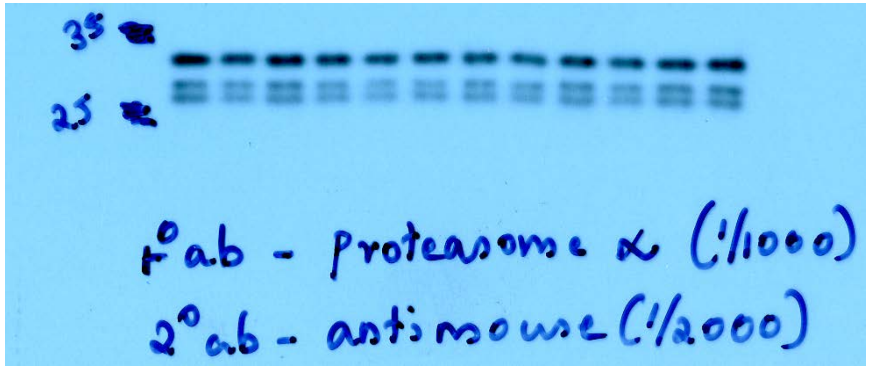

Proteasome α NUC

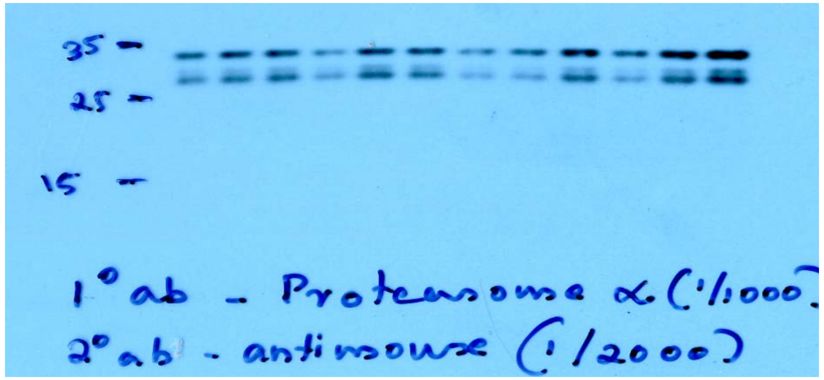

β-Actin

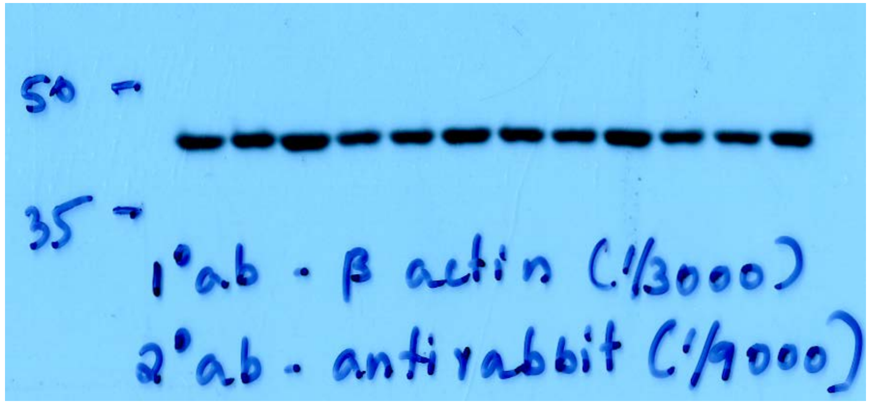

HDAC

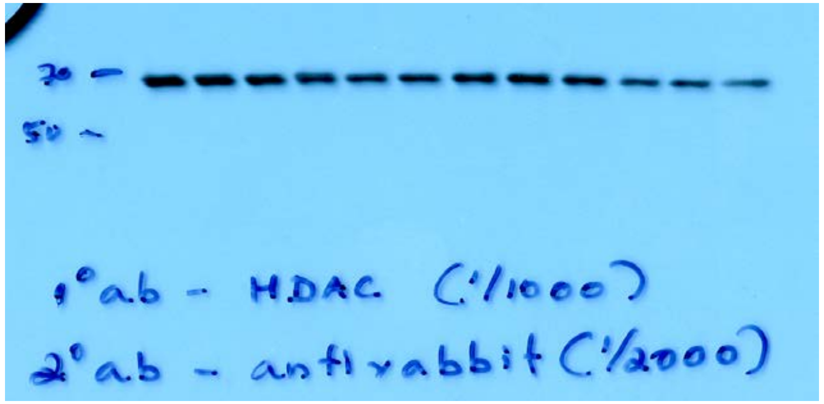

Original Western blot Images: Figure 9C
